# Lenalidomide-Associated Oral Pigmentation: A Case Report

**DOI:** 10.1007/s12105-026-01954-8

**Published:** 2026-07-24

**Authors:** André Luis Santana de Freitas, Fabiana Gomes Cardoso Pereira de Sousa, Cleverson Teixeira Soares, Rosane Rezende de Souza Giuliani, Andresa Borges Soares

**Affiliations:** 1 Department of Stomatology, School of Dentistry, Universidade Santa Cecília -UNISANTA, Santos, São Paulo, Brazil; 2 Department of Surgery, Stomatology, Pathology, and Radiology, Bauru School of Dentistry, Universidade de São Paulo, Al. Dr. Otávio Pinheiro Brisolla, 9-75, Caixa Postal 73, Bauru, São Paulo, SP 17043-101 Brazil; 3 Lauro de Souza Lima Institute (ILSL), Bauru, São Paulo, Brazil; 4 Laboratory of Anatomical Pathology of Bauru (Anatomed), Bauru, São Paulo, Brazil; 5 Universidade de Ribeirão Preto – UNAERP, Guarujá, SP Brazil

**Keywords:** Lenalidomide, Oral pigmentation, Drug-induced pigmentation, Multiple myeloma, Histopathology

## Abstract

**Background:**

Lenalidomide is an immunomodulatory agent widely used in the treatment of multiple myeloma and is generally associated with a favorable safety profile. Although pigmentary alterations such as cutaneous hyperpigmentation and hair repigmentation have been reported, oral mucosal involvement remains poorly documented.

**Case presentation:**

An 80-year-old Black female with multiple myeloma undergoing lenalidomide therapy presented with asymptomatic brown macules on the tongue, hard palate, bilateral buccal mucosa and gingiva. Histopathological examination of an incisional biopsy on the tongue revealed many fine granular, brownish pigmentation of approximately the same size throughout all epithelial layers, as well as collagen fibers, vascular walls, neural tissue, and skeletal striated muscle. These findings represent an unusual pattern of pigment distribution not previously described in patients under lenalidomide therapy.

**Conclusion:**

This case emphasizes the need for thorough oral examination in patients undergoing lenalidomide therapy, as early recognition of drug-induced pigmentation is essential for proper diagnosis and management.

## Introduction

Multiple myeloma is a common hematologic malignancy affecting the adult population. Advances in the understanding of its pathophysiology over recent decades have led to the development of novel therapeutic approaches, significantly improving disease control and overall patient survival. While conventional treatment modalities such as chemotherapy and radiotherapy remain fundamental, immunomodulatory agents, including lenalidomide, have become integral to contemporary management strategies and have shown substantial benefits in both disease progression and patient quality of life [1, 2].

Lenalidomide is a thalidomide-derived immunomodulatory drug designed to retain therapeutic efficacy while reducing adverse effects, particularly neurotoxicity and teratogenicity [3]. Its mechanisms of action include modulation of immune responses, inhibition of neoplastic cell proliferation, and suppression of tumor-associated angiogenesis [3]. Despite its relatively favorable safety profile, lenalidomide has been associated with a spectrum of adverse effects, among which pigmentary alterations have been sporadically reported, including hair repigmentation in elderly patients and isolated cases of cutaneous hyperpigmentation [4, 5]. However, data regarding pigmentary changes involving the oral and maxillofacial region remain scarce.

Recognition of medication-related mucocutaneous alterations is essential for accurate diagnosis and appropriate clinical management. Oral pigmentation may raise concern for a wide range of differential diagnoses, including melanocytic lesions, systemic diseases, medication-induced changes, and malignant neoplasms, frequently prompting biopsy or surgical intervention [6]. In this context, we report, to our knowledge, the first documented case of oral hyperpigmentation associated with lenalidomide therapy, highlighting an uncommon adverse effect with direct relevance to oral health care providers and contributing to improved awareness and diagnostic accuracy in patients undergoing long-term immunomodulatory treatment.

## Case Report

An 80-year-old Black female patient with a history of multiple myeloma, undergoing treatment with lenalidomide (5 mg/day, administered for 21 days followed by a 7-day rest period), and with a history of zoledronic acid use, was referred to our service for evaluation of oral pigmentation. Six months after initiating lenalidomide, she reported the appearance of multiple dark macules on the tongue to her physician, who referred her to an oral and maxillofacial surgeon.

**Fig. 1:**
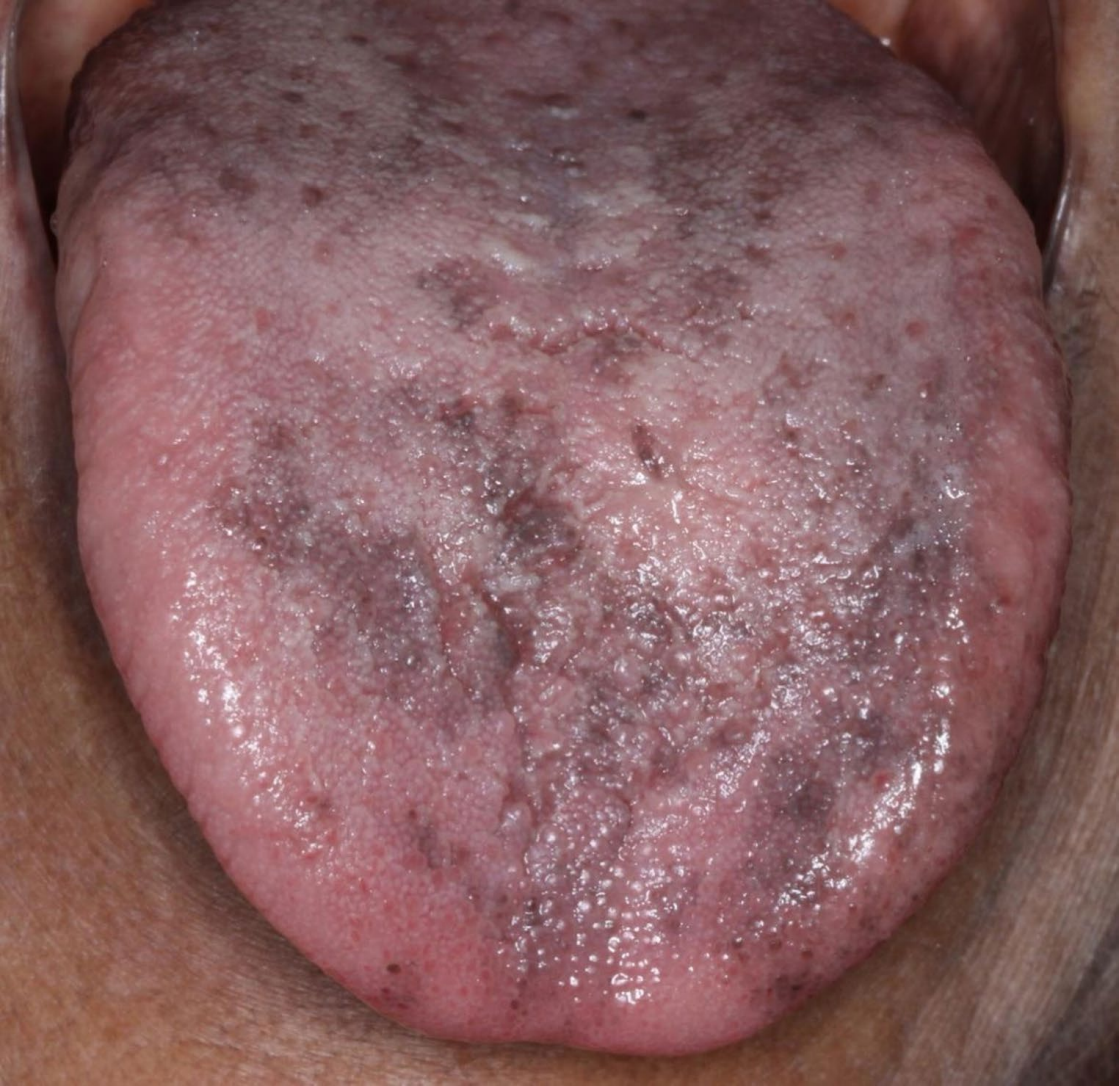
Clinical aspect of the tongue showing diffuse brown macules

On clinical examination, diffuse brownish macules observed on the bilateral buccal mucosa, hard palate, and gingiva were consistent with pre-existing racial pigmentation. In contrast, multiple brown macules on the dorsal surface of the tongue represented a recent finding that had developed after lenalidomide initiation and therefore warranted further investigation. All lesions were asymptomatic. The patient denied tobacco use and the intake of any other medications known to cause oral pigmentation (Fig. 1).

**Fig. 2:**
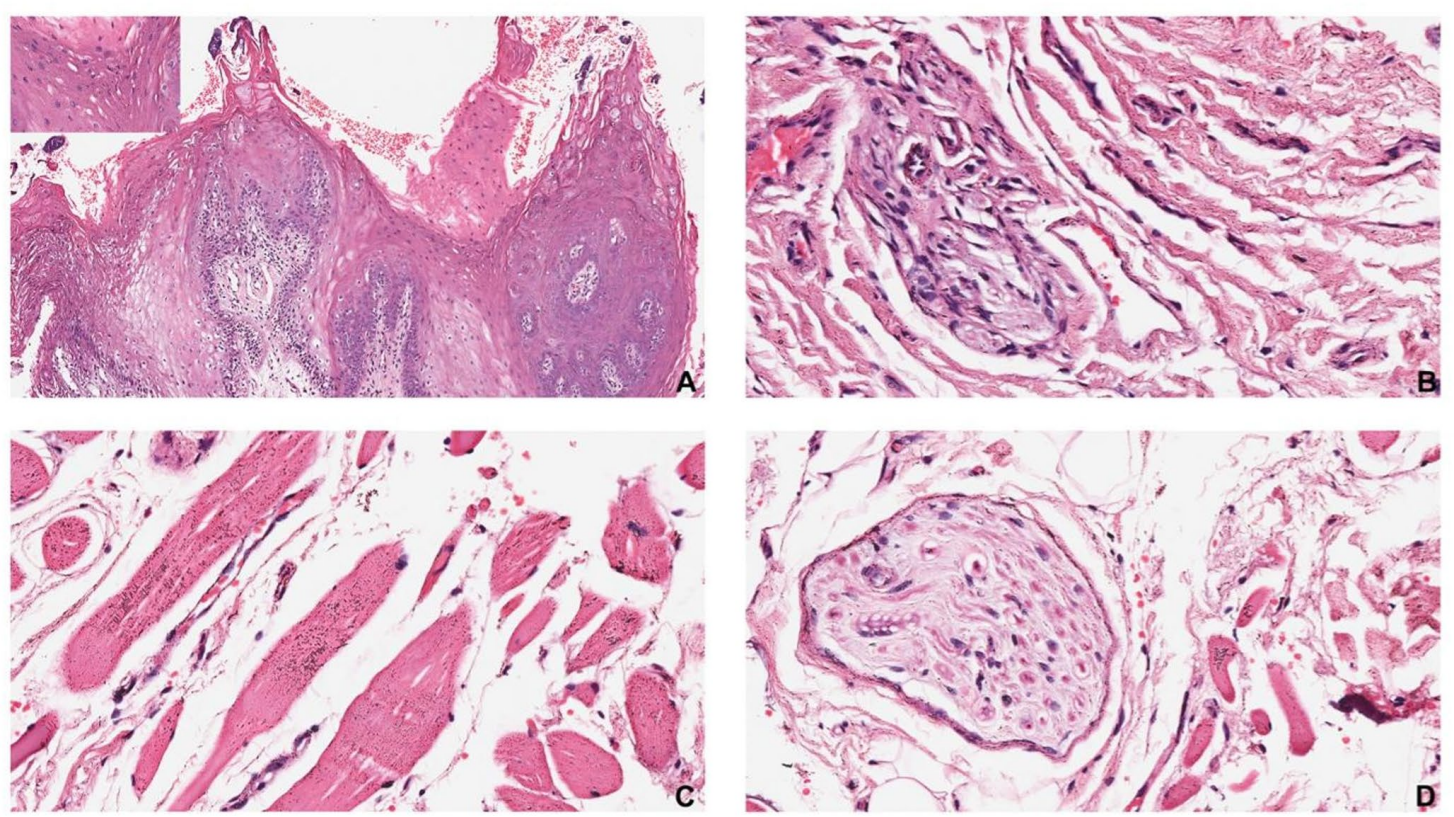
Histologic features of lenalidomide-associated pigmentation involving multiple tissue compartments. Granular, brown pigment distributed throughout all epithelial layers, inset showing pigment deposition in the superficial layers **A** deposition within collagen fibers and vascular walls **B** pigment deposition within skeletal muscle fibers **C** and neural tissue **D** *(hematoxylin and eosin stain)*

**Fig. 3:**
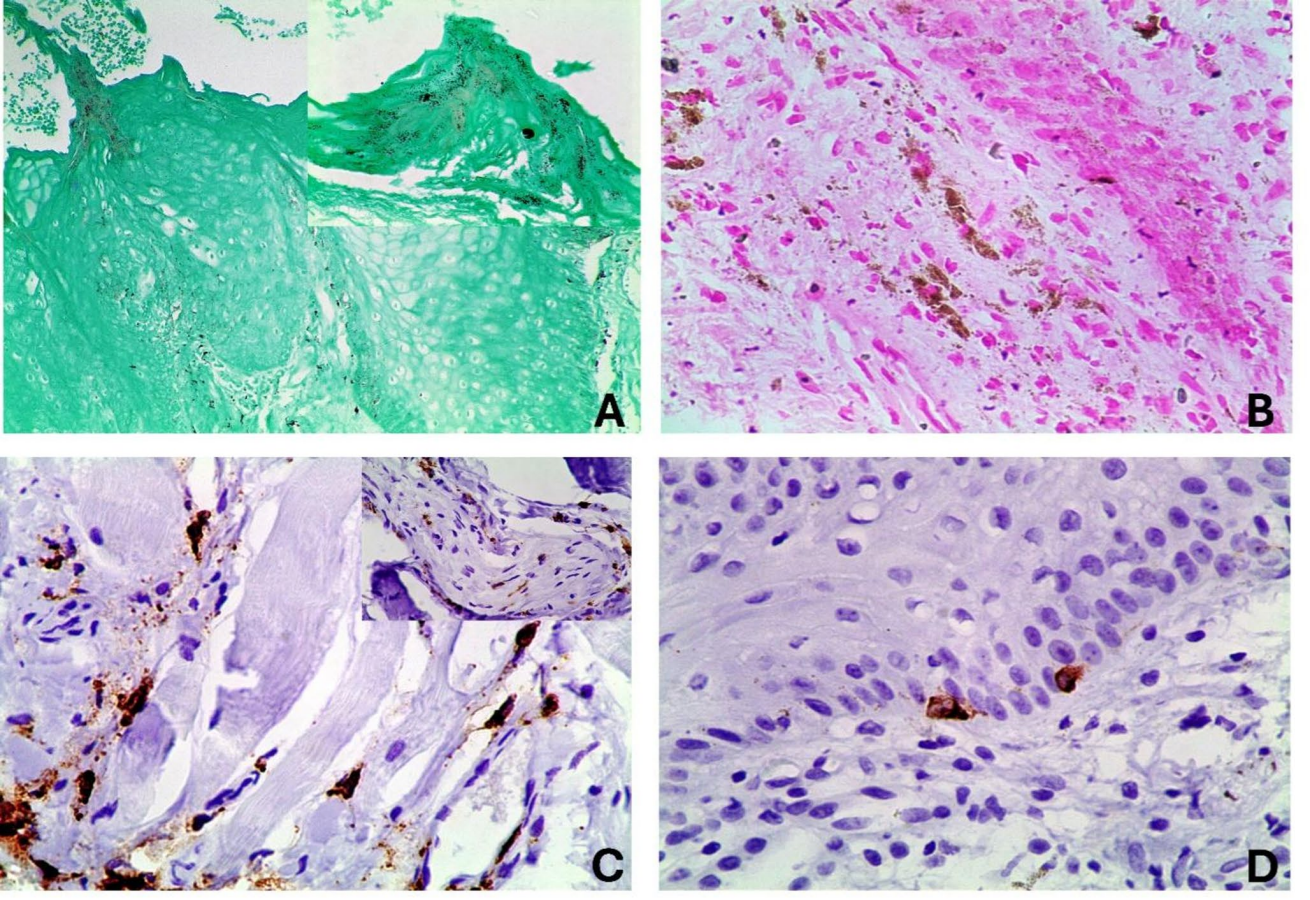
Numerous brown pigment granules diffusely distributed throughout all epithelial layers; inset showing pigment deposition in the superficial epithelial layers (Fontana–Masson stain, 40 ×) (**A)** Pigment granules negative for Perls’ Prussian blue stain (40 ×) (**B)** Numerous CD163-positive macrophages interspersed among skeletal muscle fibers and within neural tissue (inset) (**C**) Melan-A highlighting scattered basal melanocytes without evidence of melanocytic hyperplasia (**D**)

An incisional biopsy was performed on one of the recent tongue macules, with a working diagnosis of lenalidomide-induced pigmentation. Histopathological examination revealed many fine granular, brownish pigmentation of approximately the same size distributed throughout all epithelial layers and extending into the underlying connective tissue, involving collagen fibers, vascular walls, neural tissue, and skeletal striated muscle (Fig. 2). No evidence of increased melanin deposition in the basal cell layer, melanocytic hyperplasia, or associated inflammatory infiltrate was identified, although scattered melanophages were observed within the lamina propria. Histochemical staining demonstrated positive reactivity of the pigment with Fontana–Masson and negative staining with Perls’ Prussian blue. In addition, immunohistochemical analysis using Melan-A confirmed the absence of melanocytic proliferation. However, immunohistochemical evaluation with CD163 revealed numerous macrophages distributed predominantly around blood vessels, neural tissue, and skeletal striated muscle (Fig. 3).

The patient remains under lenalidomide therapy, with no changes observed in the clinical presentation to date.

## Discussion

Hyperpigmentation of the oral soft tissues has been reported as an adverse effect of several systemically administered drugs, with antibiotics, antimalarial agents, chemotherapeutic agents, hormones, and tranquilizers among the most commonly implicated [7–9]. Although cutaneous hyperpigmentation and hair repigmentation have been reported in patients with multiple myeloma receiving lenalidomide, no previous reports of oral mucosal pigmentation were identified in the literature [4, 5, 10].

In the present case, an 80-year-old Black woman diagnosed with multiple myeloma undergoing treatment with lenalidomide exhibited brown pigmentation of the tongue. Recent studies suggest that skin pigmentary alterations related to lenalidomide therapy are significantly more frequent in individuals of African descent. In a retrospective investigation, nearly half of Black patients treated with immunomodulatory agents developed some degree of cutaneous hyperpigmentation, in contrast to a minimal incidence among patients from other ethnic groups, resulting in a more than tenfold increased risk in this population [10]. These findings may indicate increased susceptibility in this population, although the biological mechanisms underlying this association remain unclear.

Although the exact pathophysiological mechanisms underlying drug-induced oral pigmentation remain incompletely understood, several hypotheses have been proposed. These include direct stimulation of melanocytes, accumulation of melanin due to impaired degradation, and/or deposition of the drug or its metabolites in pigment-rich tissues [7–9]. Recognition of these mechanisms is particularly relevant in the oral and maxillofacial region, where pigmentary changes may raise concern for melanocytic lesions and prompt invasive diagnostic procedures.

Melanogenesis, the process of melanin production by melanocytes, is tightly regulated by a balance between stimulatory and inhibitory signals. Among the well-established inhibitors of melanogenesis are several pro-inflammatory cytokines, including tumor necrosis factor alpha (TNF-α), interleukin-1 (IL-1), interleukin-6 (IL-6), and transforming growth factor beta (TGF-β). Experimental studies have demonstrated, for example, that TNF-α suppresses both the activity and expression of tyrosinase—a key enzyme in melanogenesis—through activation of the NF-κB signaling pathway [11]. In addition, IL-17 in combination with TNF-α has been shown in cellular models to suppress pigmentation-related signaling pathways and melanin production [12].

Lenalidomide is well recognized for its immunomodulatory properties, including inhibition or downregulation of the production of several pro-inflammatory cytokines. Based on this mechanism, it has been hypothesized that suppression of these negative regulators of melanogenesis may result in increased melanin synthesis or enhanced pigmentary activity, ultimately manifesting as hyperpigmentation or changes in mucosal coloration. This mechanism has been proposed to explain cases of hair repigmentation associated with lenalidomide therapy, in which reduced levels of IL-1, IL-6, and TNF-α are thought to play a contributory role [4, 5]. However, although cytokine-mediated melanogenesis may contribute to increased pigment production, it does not fully explain the unusual pattern of pigment deposition observed in this case, and the exact underlying mechanism therefore remains uncertain.

To better characterize the clinical oral pigmentation observed in this case, it was compared to the patient’s pre-existing racial pigmentation. The newly developed macules exhibited a markedly darker and more intense coloration than the patient’s physiologic pigmentation, which presented as diffuse, light to medium brown areas distributed across the oral mucosa. This distinct color difference and the localized pattern of presentation suggest that the observed pigmentation is not clinically consistent with physiologic racial pigmentation but rather represents a drug-induced phenomenon related to lenalidomide therapy.

Histologically, racial pigmentation is characterized by increased melanin deposition in basal keratinocytes and/or enhanced activity of normal numbers of melanocytes. The pigment is typically limited to the basal epithelial layers or adjacent connective tissue, sparing deeper structures [13]. In contrast, the present case shows pigmentation throughout all epithelial layers, as well as vascular walls, neural tissue, and even skeletal striated muscle. Notably, no similar pattern of deep tissue pigmentation has been reported in lenalidomide-associated cases in the reviewed literature, making this finding a potentially useful feature in the differential histopathological diagnosis.

Drug-associated hyperpigmentation of the oral mucosa may exhibit distinct microscopic patterns depending on the causative agent. Increased melanin deposition in the basal cell layer associated with pigment granules in the superficial lamina propria has been described in pigmentation related to minocycline, golimumab, and citalopram. In minocycline-associated pigmentation, histochemical findings regarding the reactivity of pigment granules to silver or iron are conflicting, whereas in golimumab-associated pigmentation, the granules react to silver but not to iron. In contrast, the absence of increased melanin deposition in the basal cell layer together with the presence of pigment granules in the reticular lamina propria has been reported in imatinib- and hydroxychloroquine-associated hyperpigmentation [7–9]. In the present case, lenalidomide-associated pigmentation did not show increased melanin deposition in the basal cell layer. Instead, the most striking morphological feature was the presence of fine pigment granules diffusely distributed throughout multiple tissue compartments, involving the full thickness of the epithelium, vascular walls, neural tissue, and, most prominently, skeletal striated muscle. Although the pigment granules were positive for Fontana–Masson and negative for iron, the overall histopathological pattern was not consistent with conventional melanin deposition. Specifically, there was no evidence of increased basal melanization or melanocytic hyperplasia. Rather, the widespread distribution of pigment within connective tissue, vascular walls, neural tissue, and skeletal muscle, together with the temporal association with lenalidomide therapy, supports the interpretation of drug-related pigmentation. Additionally, the presence of numerous CD163-positive macrophages interspersed among skeletal striated muscle fibers may suggest macrophage-mediated sequestration or processing of pigment-related material.

Although this report represents, to date, a novel finding, certain limitations must be acknowledged. The absence of previously reported similar cases precludes estimation of the true frequency of this manifestation, which may be underrecognized due to the asymptomatic nature of oral pigmentation. Future studies involving larger case series and focused investigation of lenalidomide’s effects may help confirm the morphological findings observed in the present case, as well as further elucidate the mechanisms underlying this pigmentation.

In conclusion, this report describes a case of tongue hyperpigmentation associated with lenalidomide therapy in a patient with multiple myeloma. Accurate diagnosis relies on careful clinical examination, detailed medical history, and histopathological evaluation to distinguish drug-induced pigmentation from other pigmented lesions. These findings highlight the importance of routine oral assessment in patients receiving immunomodulatory agents and reinforce the role of dentistry in recognizing medication-related mucosal alterations.

## Data Availability

No datasets were generated or analysed during the current study.
